# Study on the Fatigue and Durability Behavior of Structural Expanded Polystyrene Concretes

**DOI:** 10.3390/ma12182882

**Published:** 2019-09-06

**Authors:** Quan You, Linchang Miao, Chao Li, Huanglei Fang, Xiaodong Liang

**Affiliations:** Institute of Geotechnical Engineering, Southeast University, Nanjing 210096, China

**Keywords:** structural expanded polystyrene (EPS) concrete, long-term cyclic loading, damping ratio, dynamic elastic modulus, sulfate resistance

## Abstract

The fatigue and durability characteristics of structural expanded polystyrene concrete (EPS) are especially important when it was applied for structural elements in long-term service. In order to study the fatigue and durability behavior of structural EPS concrete, the long-term cyclic loading experiments and wetting–drying (W-D) cyclic experiments were conducted, respectively. The structural EPS concrete was found to have a relatively large damping and a fairly low dynamic elastic modulus under long-term cyclic load, which illustrated that it had a better energy absorption effect and toughness than plain concrete of the same strength level. Even if fine cracks appeared during the cyclic loading process, the relevant dynamic performance remained stable, which indicated that the structural EPS concrete had superior fatigue stability. In W-D cyclic experiments, the structural EPS concrete exhibited superior sulfate resistance. During the erosion test process, there was a positive correlation between the mass change and the evolution of the compressive strength of the structural EPS concrete, which indicated that Δm_B_ could be substituted for Δf to evaluate the degree of the structural EPS concrete eroded by sulfate attack. The study focuses on the fatigue performance and sulfate resistance of structural EPS concrete and is of important engineering value for promoting practical long-term operations.

## 1. Introduction

EPS concrete has been widely used in civil engineering structures for insulating layers or blocks [1], semi-structural or structural elements [2], sub-base of pavements, road barriers, floating marine structures, protective layer, and military structures for energy absorbing [3,4,5]. Structural EPS concrete, with design strengths between 21 and 35 MPa [6], has become a focused group of EPS concrete for the increasing strength demands of construction projects. Experimental researches on EPS concrete began in 1972, and were mainly focused on the mixing process, fluidity, and strength [7,8,9]. Actually, properties, including fatigue performance and durability, should be considered, especially when EPS concretes are designed in practical applications for long-term service.

Researches on the dynamic properties of EPS concrete were mainly focused on dynamic compressive/tensile performance [10,11] and energy absorption properties under drastic instantaneous impact [4,5]. Actually, EPS concrete could be designed to endure cyclic loadings, such as traffic loads, and its dynamic properties would not be the same as those measured in other conditions [12]. Hence, it is important to study the dynamic properties of EPS concrete under cyclic loadings. It was found that the larger the applying dynamic cyclic load is, the more obviously the compressive strength changes [13]. However, the accumulative vibration number reported in this paper was 100,000 times. Cyclic loading experiments with far more vibration numbers should be conducted to study the fatigue performance of EPS concrete under long-term cyclic loading.

Durability is another factor influencing the long-term service of EPS concretes, while a limited number of studies concerning the durability of EPS concrete have been reported currently. It seemed that a dispute does exist over the durability of EPS concretes. Owing to the micro-cracks formed by the shrinkage of EPS aggregate, EPS concrete containing higher volumes of EPS aggregate was reported to present higher moisture migration and absorption capacity [2], which could result in a worse durability of the concrete [14]. Adversely, other test results showed that the effect of chemical attack, like chloride penetration, was reduced with the increase of the EPS volume, as a result of the lower cement contents in EPS concretes [15]. Visual assessment and weight measurements on EPS concretes in the 10% sodium sulfate solution also illustrated that EPS concrete was nearly unaffected by sulfate attack at periodic intervals for the five-week duration [16], suggesting that EPS concretes had better sulfate resistance than plain concrete. Therefore, it is valuable to study the performance of EPS concrete under sulfate attack, which is one of the most serious durability problems [17]. In fact, to prepare EPS concretes for sulfate attack tests, the influence of sulfate attack on cementitious materials should be considered because the concrete matrix is a non-negligible component of the EPS concrete. Portland cements with low contents of C_3_A [18], as well as the addition of supplementary cementitious materials, such as fly ash [19], silica fume [20,21], and limestone powder [22], are more preferred for bearing sulfate attack. Reducing the water:binder ratio to 0.40 or a lower value has also been recommended to enhance the resistance of cementitious materials during physical sulfate salt attack [18]. Hence, apart from EPS beads’ incorporation, it is of importance to design the matrix mixture proportions of EPS concretes carefully, especially when concretes are exposed to a relatively severe sulfate attack environment.

In this study, structural EPS concretes with a constant mixture proportion and EPS incorporation ratio were prepared to ensure the workability and strength. The long-term cyclic loadings with different frequencies (5, 10 Hz) and amplitudes of the bias force (30, 40 kN) in a total of 22,000,000 vibration times were conducted by a MTS 810 universal testing machine. The fatigue behavior, including dynamic stress–strain hysteresis curves, corresponding damping ratio, and dynamic elastic modulus at different cyclic numbers, was then analyzed. Also, the sulfate resistance of structural EPS concretes was investigated by W-D cyclic experiments. The compressive strength and mass of structural EPS concrete during the whole experiment were recorded and the level of sulfate resistance was evaluated. Finally, the mechanisms of variations of mass and compressive strength were analyzed, and a positive correlation was found between the mass change and the evolution of compressive strength of the structural EPS concrete.

## 2. Materials and Experiments

### 2.1. Materials

Concretes were prepared using 42.5 Portland Cement (similar to type V in ASTM C150/C150M [23]) and fly ash of Class F in ASTM C618 [24]. Portland cement of type V and fly ash of Class F were recommended to enhance the sulfate resistance of the cement composition [17,19,25]. As for the cement used in the experiment, C_3_A and C_4_AF + 2C_3_A contents were 1.81% and 15.87%, respectively, well below the 5% and 25% limits of ASTM C150/C150M. The properties of the fly ash are given in Table 1.

Crushed basalt gravel, with two different sizes labeled type A and B, was used as coarse aggregate. The particle size distribution of type A and B ranged from 5 to 15 mm and 15 to 25 mm, respectively. Yangtze River sand, with a specific gravity of 2.59 and water absorption of 0.6%, was used as fine aggregate. In addition, the commercially available EPS beads, with a particle size ranging from 2 to 3 mm, were used as lightweight aggregates. A commercial high-performance polycarboxylic-based superplasticizer (SP) with a density of 1080 kg/m^3^ and a solid content of 22% was used to improve the fluidity of the mix. Also, a commercial powdery viscosity-modifying admixture (VMA) and another commercial admixture polymer emulsion (PE) were employed to increase the viscosity of the cement paste and the hydrophilicity of the EPS beads, respectively.

### 2.2. Mixture Proportion and Sample Preparation

The mixture proportion is listed in Table 2, which is a reasonable mixture proportion that could inhibit the segregation and guarantee the strength and workability of the structural EPS concrete [26]. The whole plain concrete was replaced by EPS aggregates with 30% by volume to guarantee that the compressive strength and the slump of the produced structural EPS concretes were higher than 30 MPa and 200 mm, respectively. SP with a dosage of 1.5% by weight of the total cementitious materials (TCM) would enhance the fluidity of the concrete paste with a water:binder ratio of 0.29 designed to the maximum extent. It was found that when the dosage of PE was 1.0% by weight of TCM, PE could approximately increase the hydrophobic ability of EPS at full capacity [13].

The concrete mixtures were mixed in a recommended subsequence [27], and the whole production process was in accordance with ASTM C192 [28]. The concrete mixtures were casted into cubic specimens with an edge length of 100 mm. After casting for 24 h, the specimens were demolded and cured in the standard curing room at temperature of 23 ± 2 °C and relative humidity not less than 95% according to ASTM C511 [29] until the age of the corresponding tests. Specimens with the same mixture proportion were prepared for the long-term cyclic loading experiment and the sulfate attack experiment.

### 2.3. Long-Term Cyclic Loading Experiment

**Figure 1:**
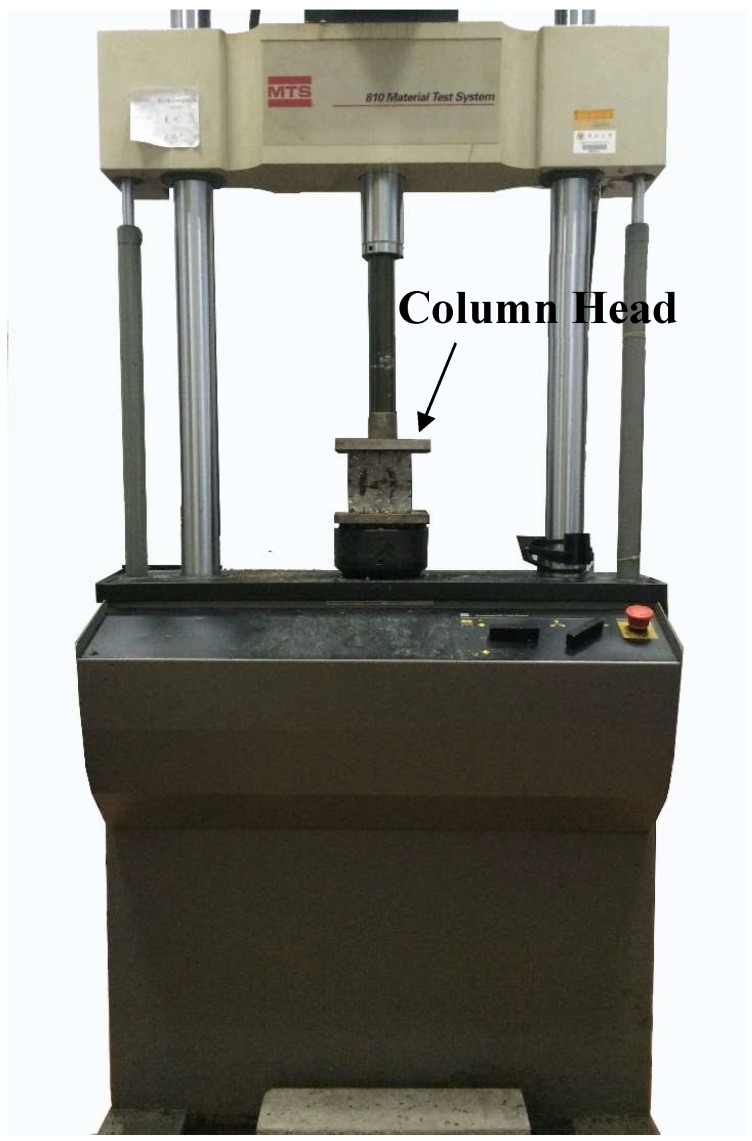
Cyclic loading test apparatus.

After curing for 28 days, the long-term cyclic loading test was carried out on the specimens by the material test system (MTS) 810 (MTS Systems Corporation, Eden Prairie, MN, USA), as shown in Figure 1. The sinusoidal load was applied on the specimen for the long-term cyclic loading test, and 25 data points were collected each cycle. The damping ratio and dynamic elastic modulus of structural EPS concrete were then measured and analyzed based on the hysteresis loop of the long-term cyclic loading tests.

The loading frequencies of the cyclic loading were set at 5 and 10 Hz, and the amplitudes of the bias force were taken as 30 and 40 kN, respectively, to model the dynamic load action on the upper surface of the ballast bed [13]. The cumulative vibration was 22,000,000 times (a total of 708 h and 20 min). 

The long-term cyclic loading test underwent four stages as tabulated in Table 3. By comparing the dynamic properties of structural EPS concretes between stage Ⅰ and Ⅱ, the effect of amplitudes of the bias force could be observed, and the comparison between stage Ⅱ and Ⅲ could help verify the effect of frequency. When the accumulated vibration time was around 15.7 million, fine cracks appeared on the face of the specimen, so the amplitudes of the bias force were adjusted to 30 kN in stage Ⅳ.

To verify whether the variations of the damping ratio and dynamic elastic modulus during the long-term cyclic loading test were caused by the differences of the frequencies and loading amplitudes instead of the loading history, additional cyclic loading experiments were also conducted on another two specimens with the same mixture proportion. This experiment also consisted of four stages, and each stage had 100,000 vibration times to exclude the possibility of fine cracks as observed in the first long-term cyclic loading experiments. During the additional loading experiments, the first three stages had the same amplitudes of the bias force and loading frequency of stage Ⅰ to Ⅲ in Table 3, and stage four was with the same load amplitude and frequency of stage Ⅰ. By comparison between the first stage and the fourth stage of the additional cyclic loading experiments, whether the dynamic properties under the same loading condition remained stable after a period of loading history could be studied.

### 2.4. Sulfate Attack Experiment

#### 2.4.1. W-D Cyclic Experiment

The W-D cyclic sulfate attack was relatively severe compared to other sulfate attack conditions, because phase changes between the nardite and mirabilite were triggered [18]. The W-D cyclic sulfate attack experiment provided by GB/T 50082 [30] was applied to assess the sulfate resistance of structural EPS concrete.

**Figure 2:**
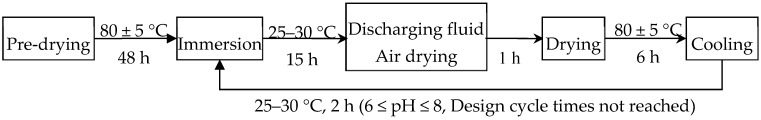
The procedure of the wetting–drying (W-D) cyclic experiment.

All samples were cured in the standard curing room until the W-D cyclic experiments were conducted. The procedure of the W-D cyclic experiment is shown in Figure 2.

Two days before the 28-day curing age, the specimens for the W-D cyclic experiment were removed from the standard curing room. The surface moisture of the tested specimen was firstly wiped, then the specimen was placed into an oven and baked at 80 ± 5 °C for 48 h. After drying, the specimens were cooled to room temperature in a dry environment.

When the pre-drying work was finished, the specimens were put into a covered box made of salt corrosion resistant material. A 20-mm spacing was maintained between adjacent specimens, and the distance between the specimen and the side wall should also be at least 20 mm. Then, the prepared 5% sodium sulfate solution was poured into the box quickly, with the final liquid level more than 20 mm above the surface of the uppermost specimen. The time for the injecting process and total immersion duration should not exceed 30 min and 15 h, respectively. The sodium sulfate solution was replaced each month to keep the pH value of the solution between 6 and 8 [30] because the sulfate attack triggered by sodium sulfate solution in this state was reported to be the severest [31]. Also, the temperature of the solution should be controlled at 25 to 30 °C during the immersion process.

After being immersed for 15 h, the solution was drained in 30 min; subsequently, the sample was air dried for 30 min. The temperature in the box was increased to 80 °C immediately after the air-drying process, and the heating process should be completed within 30 min. When the temperature reached 80 °C, it should be maintained at 80 ± 5 °C for 6 h. After the drying process, the specimens should be cooled promptly, and the surface temperature of the specimen should be cooled to 25 to 30 °C in 2 h. The total time for each W-D cycle was 24 h. If the designed cycle times were not reached, the samples were re-immersed in the sulfate solution and a new cycle from immersion to cooling continued.

#### 2.4.2. Compressive Strength Experiment and Mass Measurement

**Figure 3:**
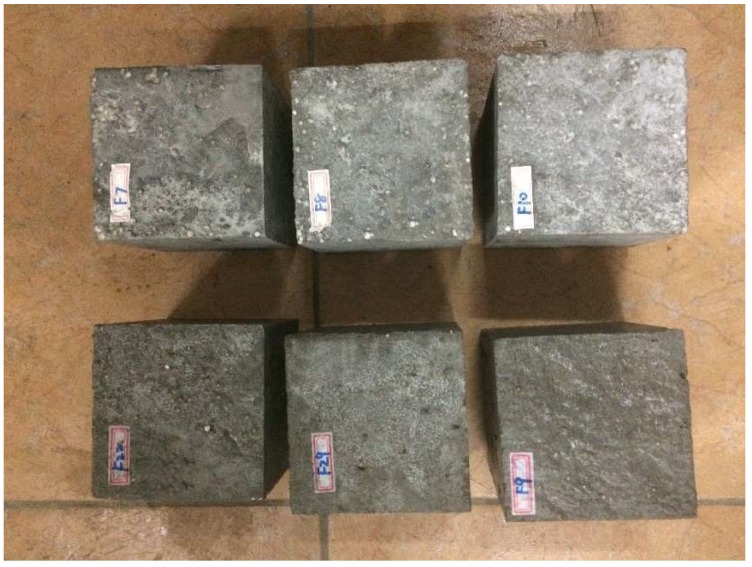
Specimens of group B and group C.

According to the specification [30], structural EPS concretes prepared in this study were classified into three groups for different uses. Group A was used to measure the initial strength of the structural EPS concrete after 28 days of standard curing. Specimens of group B underwent sulfate attack and exposed to W-D cyclic environment, and specimens of group C were treated with distilled water for comparison with group B. As shown in Figure 3, specimens F7, F8, and F10 in the first row were examples after sulfate attack (group B), while specimens F22, F29, and F9 in the second row were cured in distilled water (group C).

When corresponding W-D cycles were finished, the compressive strength and mass of structural EPS concretes of group B and group C were measured for compressive strength simultaneously. The average weight of all specimens was measured and calculated before the compressive strength experiment.

The sulfate-resistant coefficient of compressive strength for structural EPS concrete was calculated as follows:

(1) $$K_{f}=f_{Bn}/f_{Cn}\times 100\%,$$

where $f_{Bn}$ is the average compressive strength of structural EPS concrete of group B; $f_{Cn}$ is the average compressive strength of structural EPS concrete of group C measured at the same time when the corresponding structural EPS concretes of group B were measured. All dimensions are in MPa and accurate to 0.1 MPa.

Six sulfate resistance levels, including KS30, KS60, KS90, KS120, KS150, and above KS150 [30], were set to evaluate the sulfate resistance of concretes. These six sulfate resistance levels of structural EPS concrete could be obtained by the maximum number of W-D cycles when the sulfate resistant coefficient, *K_f_*, was reduced to no less than 75%. For example, if *K_f_* for the 60th W-D cycle was more than 75%, and that for 90th W-D cycle was less than 75%, then the sulfate resistance level would be KS60. Because the sulfate resistance level of the structural EPS concrete was uncertain, *K_f_* was measured every 30 W-D cycles until *K_f_* was below 75% from the 30th cycle. 

## 3. Experimental Results and Discussions

### 3.1. Fatigue Performance of Structural EPS Concrete

#### 3.1.1. Dynamic Stress-Strain Hysteresis Curves

**Figure 4:**
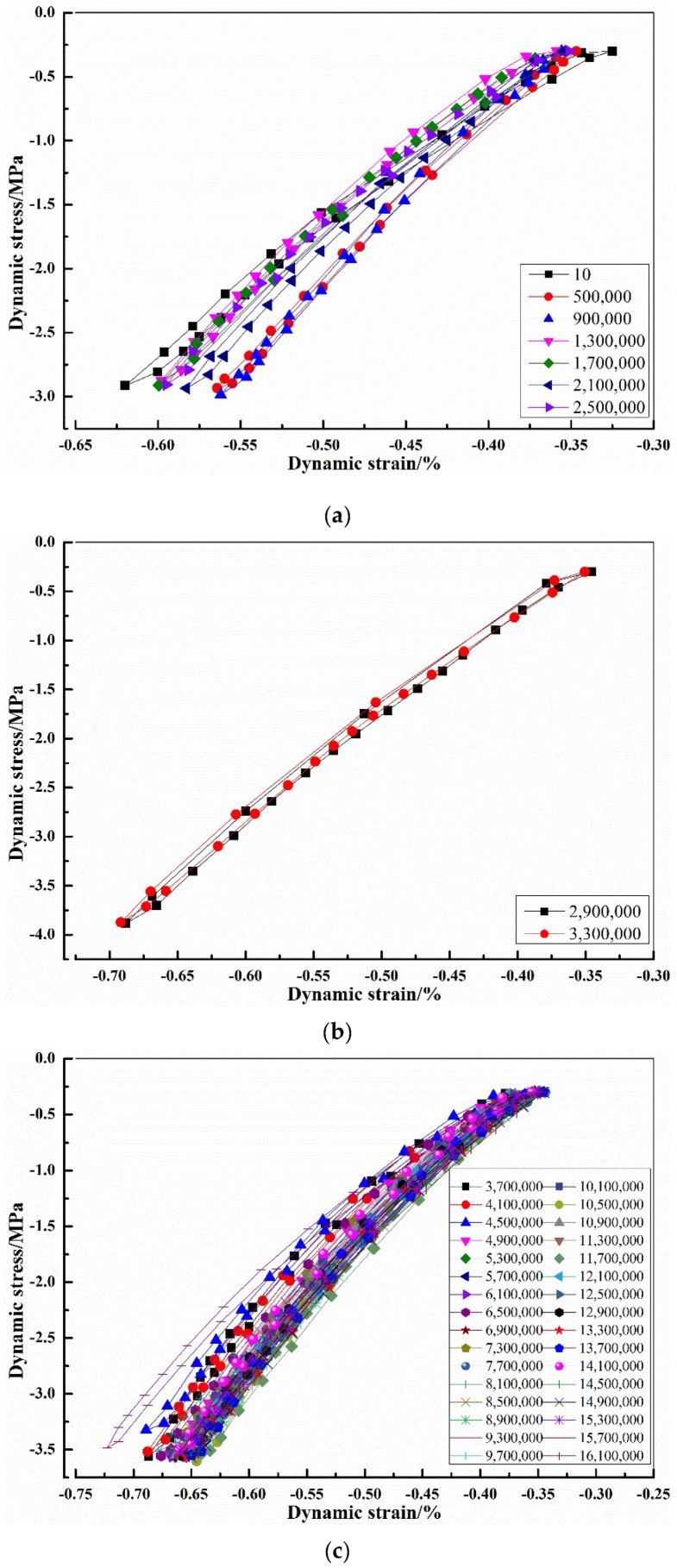

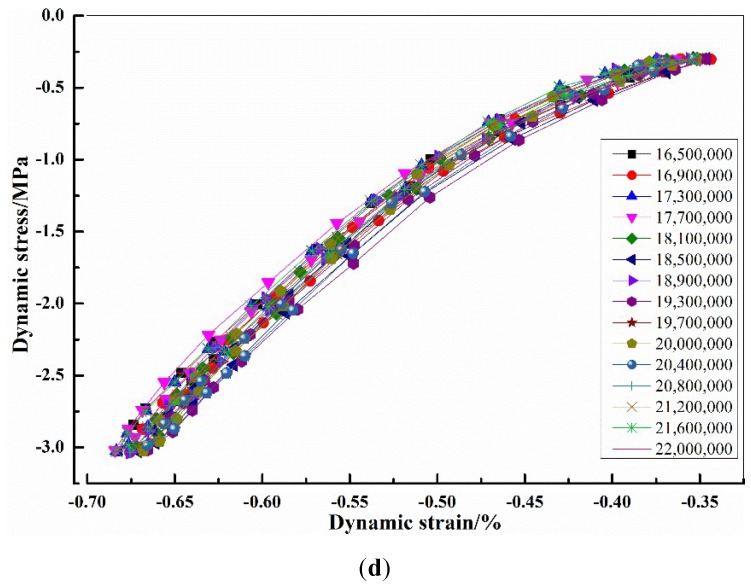
Dynamic stress–strain hysteresis curves of structural EPS concrete: (**a**) 0–2,500,000 times; (**b**) 2,500,000–3,500,000 times; (**c**) 3,500,000–16,350,000 times; (**d**) 16,350,000–22,000,000 times.

Owing to a large amount of data, 10 groups of hysteresis curve data were selected and averaged every 400,000 vibrations before further processing. In the course of 22,000,000 times of cyclic loading, the corresponding stress levels and frequencies were not the same. Therefore, the dynamic stress–strain hysteresis curves were drawn by stages for analysis. The dynamic stress–strain hysteretic curves of 0–2,500,000, 2,500,000–3,500,000, 3,500,000–16,350,000, and 16,350,000–22,000,000 times are respectively shown in Figure 4a–d.

From Figure 4a, it could be found that under the same load and frequency, the hysteresis loop corresponding to the 10th vibration had a larger dynamic strain difference for one cycle compared with those for the 500,000th and 900,000th vibration. The main reason was that the vibration had not reached a relatively stable state until the 10th cycle.

**Figure 5:**
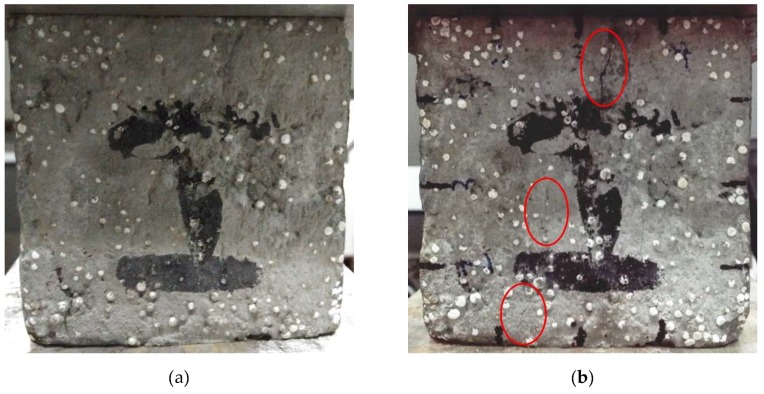
The face of structural EPS concrete before and after micro-cracks appeared: (**a**) 15,500,000 times; (**b**) 16,350,000 times.

It can be seen from Figure 4c that in the dynamic stress–strain hysteresis curves corresponding to 15.7 million times, the strain with the same stress level was obviously larger. Referring to the test records, the specimens had fine cracks at about 15.7 million vibrations as shown in Figure 5. Figure 5a is the face of the specimen for 15.50 million vibrations before micro-cracks appeared, and Figure 5b is the specimen at 16.35 million vibrations after micro-cracks were observed. It could be observed from Figure 5 that some micro-cracks appeared on the face of the structural EPS concrete. The existence of fine cracks made the curve appear unstable for a while, but the specimens were stabilized again when the vibrations continued, and the dynamic stress–strain curve also tended to be consistent.

Figure 4 illustrates that under the same load and frequency, the dynamic stress–strain curve of structural EPS concrete basically overlapped with each other, indicating that the structural EPS concrete had good stability even under a dynamic load.

#### 3.1.2. Damping Ratio

The damping ratio, as one of the dynamic characteristics of the structure, is an important indicator that could reflect the damping capacity. The damping ratio often means the ability to absorb and attenuate vibrational energy. The damping ratio of structural EPS concrete could be calculated by the dynamic stress–strain hysteresis curve, and the corresponding damping characteristics could then be analyzed.

**Figure 6:**
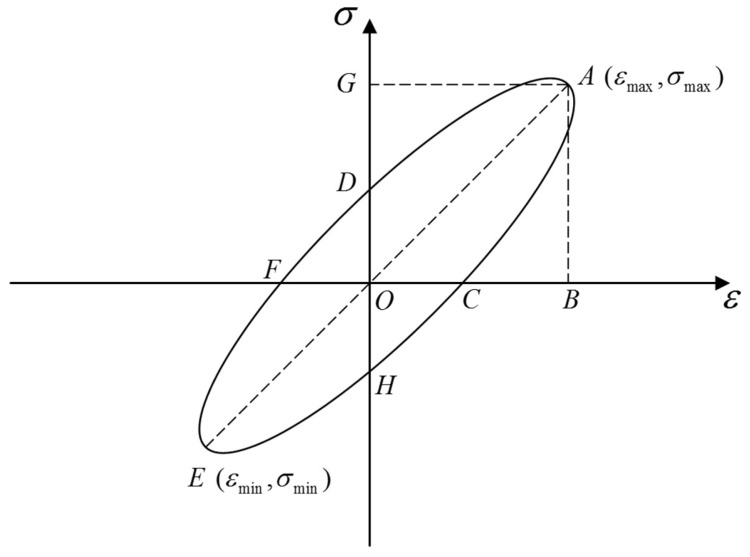
Standard hysteresis loop.

Figure 6 shows a standard hysteresis loop curve. The total energy in one cycle is equal to the area of the triangle *OAB*, so the damping ratio could be calculated as follows:

(2) $$\lambda =\frac{1}{4\pi }\frac{A_{0}}{A_{T}},$$

where *A*_0_ is the area of the hysteresis loop, and *A_T_* is the area of the triangle, *OAB*.

The area of the triangle *A_T_* could be obtained according to Equation (3):

(3) $$A_{T}=\frac{1}{8}(\sigma_{max}-\sigma_{min})(\varepsilon_{max}-\varepsilon_{min}),$$

where *ε_max_*, *σ_max_*, *ε_min_*, and *σ_min_* are the dynamic strains and dynamic stresses at points *A* and *E* in Figure 6, respectively.

In a two-dimensional surface, the area of the triangle pinched by two vectors is equal to half of the vector module of the cross product. Therefore, the area of hysteresis loop *A*_0_ could be obtained by substituting the 25 data points in each cycle subsequently in the clockwise direction into Equation (4), and the damping ratio could be further calculated:

(4) $$A_{0}=-\frac{1}{2}(\left|\begin{array}{cc} \varepsilon_{1} & \sigma_{1}\\ \varepsilon_{2} & \sigma_{2} \end{array}\right|+\left|\begin{array}{cc} \varepsilon_{2} & \sigma_{2}\\ \varepsilon_{3} & \sigma_{3} \end{array}\right|+\cdots +\left|\begin{array}{cc} \varepsilon_{24} & \sigma_{24}\\ \varepsilon_{25} & \sigma_{25} \end{array}\right|+\left|\begin{array}{cc} \varepsilon_{25} & \sigma_{25}\\ \varepsilon_{1} & \sigma_{1} \end{array}\right|)\;,$$

where $\varepsilon_{i}$ and $\sigma_{i}$ are the dynamic strains and dynamic stresses at points labeled *i*, respectively.

In the additional loading experiments, the calculated damping ratios of stage four and one were both around 0.0200, which indicated that the damping ratio and dynamic elastic modulus of structural EPS concrete remained nearly invariable after a long time of the cyclic loading process. Hence, further analysis could be carried out on the variation trends of dynamic properties with the change of the loading frequency and amplitude.

**Figure 7:**
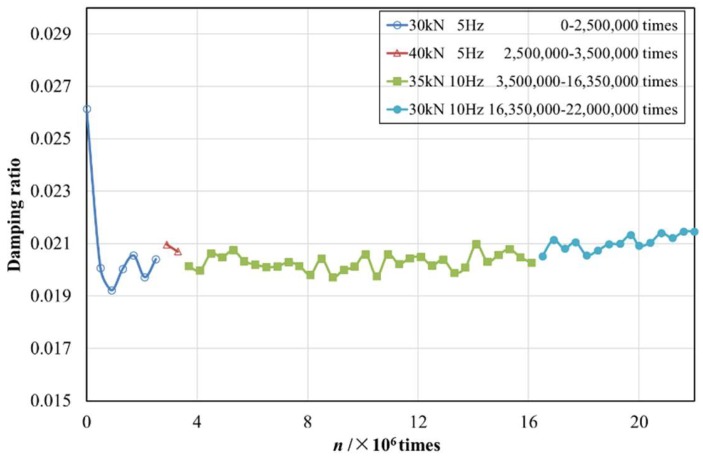
Damping ratio–cyclic number (*n*) curve of structural EPS concrete.

The dynamic stress–strain hysteresis curve of structural EPS concrete every 400,000 vibrations was processed and the corresponding damping ratio was obtained. As shown in Figure 7, the vibration was divided into four stages according to the load and frequency.

The whole structural EPS concrete could be viewed as a kind of material, then the mechanism of forming damping under dynamic loading mainly included three aspects: The damping part of the concrete itself; the damping part of the polystyrene material; the structural damping part of the interface of the concrete chamber and polystyrene particles. The last two parts were also the reasons why the damping mechanism of the structural EPS concrete differed from that of ordinary concrete.

It was found that the larger the vibration load is, the greater the damping ratio of EPS concrete will be [13]. Compared with the average damping ratio before 15.7 million times in Figure 7, the average damping ratio of stage Ⅱ (0.0208) at a load of 40 kN was larger than the average damping ratio of stage Ⅰ at a load of 30 kN (0.0200), which was basically consistent with existing studies. With the increment of frequency from 5 Hz (stage Ⅱ) to 10 Hz (stage Ⅲ), the damping ratio increased slightly.

Under cyclic loading, the chamber of the concrete specimen where the polystyrene particles resided was repeatedly compressed and expanded, causing repeated friction between the particles and the concrete chamber, as well as friction in the chamber itself. When the cyclic load amplitude was relatively large, the friction between the components was relatively strong, resulting in an increase in energy consumption; also, the particles corresponding to the larger cyclic loads were more deformed, and the polystyrene particles could not be timely adjusted to the original size, making the friction with the concrete chamber more severe [32]. Therefore, the load increased, and the damping ratio of structural EPS concrete increased. Because the damping ratio reflects the ability to reduce kinetic energy in the interior of the concrete, the bigger the vibration load was, the more vibration energy the structural EPS concrete could attenuate and absorb. 

Although the load and frequency were not the same in different stages, the damping ratio was approximately stable at 0.0205 for structural EPS concrete with an EPS incorporation ratio of 30%. The damping ratio of ordinary concrete with a strength grade of C30 was approximately 0.0090 to 0.0147 [33]. It was reported that the damping ratio of plain concrete of the same mixture proportion without EPS particles was stable at about 0.0128 under the cyclic loading of a 40 kN amplitude and 5 Hz frequency [13]. It can be seen that the structural EPS concrete prepared in this experiment had a high damping ratio (above 0.020), indicating that it had a better energy absorption effect. It could be further observed from Figure 7 that even if the specimen had micro-cracking (around 15.7 million cycles), the damping ratio was still relatively stable (basically slightly larger than 0.0200).

#### 3.1.3. Dynamic Elastic Modulus

To study the mechanical response under dynamic load, apart from factors under static action, the time, size, frequency, and repetition effects of the load should also be considered, which made the dynamic response have a certain stress dependence [34]. Correspondingly, the dynamic elastic modulus of EPS concrete was not exactly the same under different environmental conditions. In this study, the dynamic elastic modulus was measured under the dynamic load similar to the subway vibration.

In cyclic loading tests, the dynamic elastic modulus of each hysteresis loop was the slope value of the vertex connection, which can be calculated according to Equation (5):

(5) $$E_{d}=(\sigma_{max}-\sigma_{min})/(\varepsilon_{max}-\varepsilon_{min}).$$

**Figure 8:**
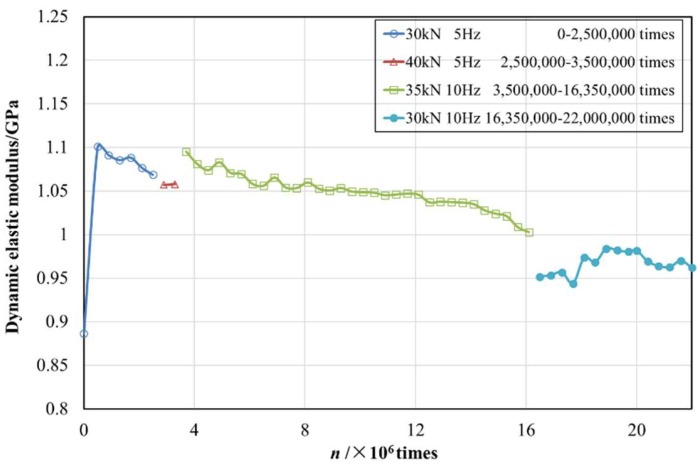
Dynamic elastic modulus–cyclic number (*n*) curve of structural EPS concrete.

Similar to the processing method of the damping ratio, the dynamic elastic modulus of different vibration times is shown in Figure 8. 

From Figure 8, it could be found that when the 10th cyclic load was applied, the specimen did not reach a stable state; thereafter, the dynamic elastic modulus remained basically unchanged (about 1.07 GPa), indicating that the dynamic elastic modulus of the structural EPS concrete specimen had good durability under long-term cyclic load. When the plain concrete grade was above C30, the static elastic modulus was about 30 GPa. According to the relationship between the static and dynamic modulus of the plain concrete [35,36], the dynamic elastic modulus would be 36 to 50 GPa. The strength of the structural EPS concrete specimens prepared in this study fulfilled the strength level of C30, and the dynamic elastic modulus was a magnitude order less than that of plain concrete of the same strength level. It was reported that the higher the dynamic elastic modulus of concrete was, the greater brittleness and worse crack resistance of concrete would be [12]. Thus, it could be seen that the structural EPS concrete had stronger toughness and superior fatigue stability.

Comparing the cyclic loading of 0–2.5 million times (stage Ⅰ) with 2.5–3.5 million cycles (stage Ⅱ), given the same frequency, the increase of the load amplitude led to a slight decrease in the dynamic elastic modulus. To the contrary, the increment of frequency from 5 Hz (stage Ⅱ) to 10 Hz (stage Ⅲ) induced growth of the dynamic elastic modulus. This was due to the fact that with the increase of the load amplitude, new cracks would be generated inside the EPS concrete, and the original micro-cracks would expand, then the deformation would increase and the corresponding dynamic elastic modulus would decrease.

From 3.5 million cycles to 16.35 million cycles, it was found that even with the same load and frequency, the dynamic elastic modulus of structural EPS concrete had an explicit tendency of gradually decreasing with the accumulated cycles. Accompanied by the propagation of micro-cracks and the generation or pooling of new cracks, an increase in deformation and a decrease in the dynamic elastic modulus were thus monitored. In particular, micro-cracks appeared on the face of the specimen near the 15.7 million cycles, and the value of the dynamic elastic modulus oscillated, but then gradually stabilized. After 15.7 million cycles, the dynamic elastic modulus only slightly decreased to a value between 0.944 and 0.992 GPa.

### 3.2. Sulfate Resistance of Structural EPS Concrete

The variation of compressive strengths and mass during the whole process of sulfate attack were investigated to evaluate the sulfate resistance of structural EPS concrete.

#### 3.2.1. Sulfate Resistance Level of Structural EPS Concrete

The values *f_Bn_* and *f_Cn_* measured at every 30 cycles ate tabulated in Table 4, and corresponding *K_f_* was also calculated in accordance with Equation (1). 

It was found that *K_f_* was 94.1% when the cyclic number reached 150, which meant that the sulfate resistance level of structural EPS concrete was over KS150, the maximum sulfate resistance level regulated by the specification [30]. *K_f_* was not below 75% until the 210th W-D cycle came, so the sulfate attack experiment was carried out to the cyclic number of 210.

#### 3.2.2. Variation of the Mass of Structural EPS Concrete

The variation of mass (Δ*m*) was defined according to Equation (6):

(6) $$\Delta m=(m_{n}-m_{0})/m_{0},$$

where $m_{0}$ is the initial average weight of EPS concrete when the concrete is taken out of a standard curing room at the 28-day curing age; $m_{n}$ is the average weight of EPS concrete of group B experiencing *n* W-D cycles or average weight of EPS concrete of group C at the time of *n* cycles. All dimensions are in grams.

**Figure 9:**
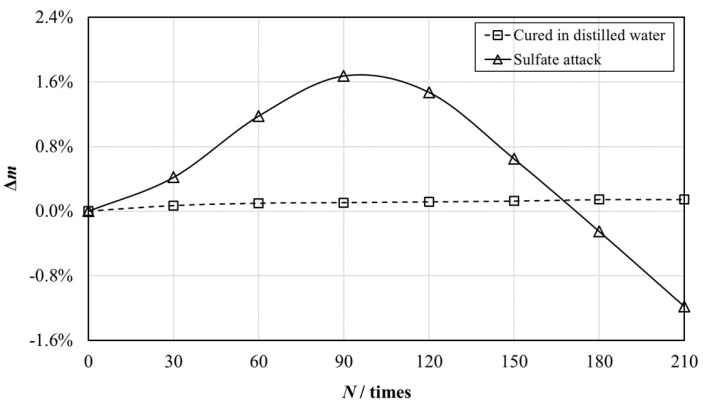
The evolution of mass of structural EPS concretes of group B and C with different numbers of W–D cycles (*N*).

The evolution of the mass of structural EPS concretes of group B and C after different numbers of W–D cycles is illustrated in Figure 9. As illustrated, the mass of the samples of group B increased in the first 90 W–D cycles and then decreased with the number of W–D cycles, while the weight of specimens of group C climbed slowly with the increased curing days. 

After standard curing for 28 days, hydration reactions of components in structural EPS concretes were still in progress, which made the mass of structural EPS concretes of group C increase steadily. 

Besides hydration reactions, the weight of structural EPS concrete of group B was also influenced by sulfate attack. Sulfate attack could be primarily classified into two modes: The physical attack and chemical attack. The physical attack was owing to cycles of conversion between thenardite (Na_2_SO_4_) and mirabilite (Na_2_SO_4_⋅10H_2_O) [37], and the chemical attack was induced by the formation of ettringite and gypsum [17], arising from reactions between sulfate ions and cement hydration products or cement ingredients. In the W-D cyclic experiment, the consecutive precipitation of mirabilite and gypsum were the main products [38,39], which resulted in the expansion of EPS concretes. These expansive products could be used to explain the variation of the mass of structural EPS concretes of group B. In the first 90 W-D cycles, the products filled up the pores in the specimens, and the weight increased as a result. Subsequently, the continuous attack resulted in the disruption of EPS concretes, and the weight started to reduce. 

As depicted in Figure 9, the peak value of the variation of the mass of structural EPS concretes under sulfate attack in this study was 1.68%, which is much smaller than that of plain concrete (12%) under the same experiment conditions [40]. It was owing to the incorporation of EPS beads and fly ash, low water:binder ratio, and the choice of cement type designed in this study that made the structural EPS concrete have superior sulfate resistance and less weight variation than ordinary concrete.

**Figure 10:**
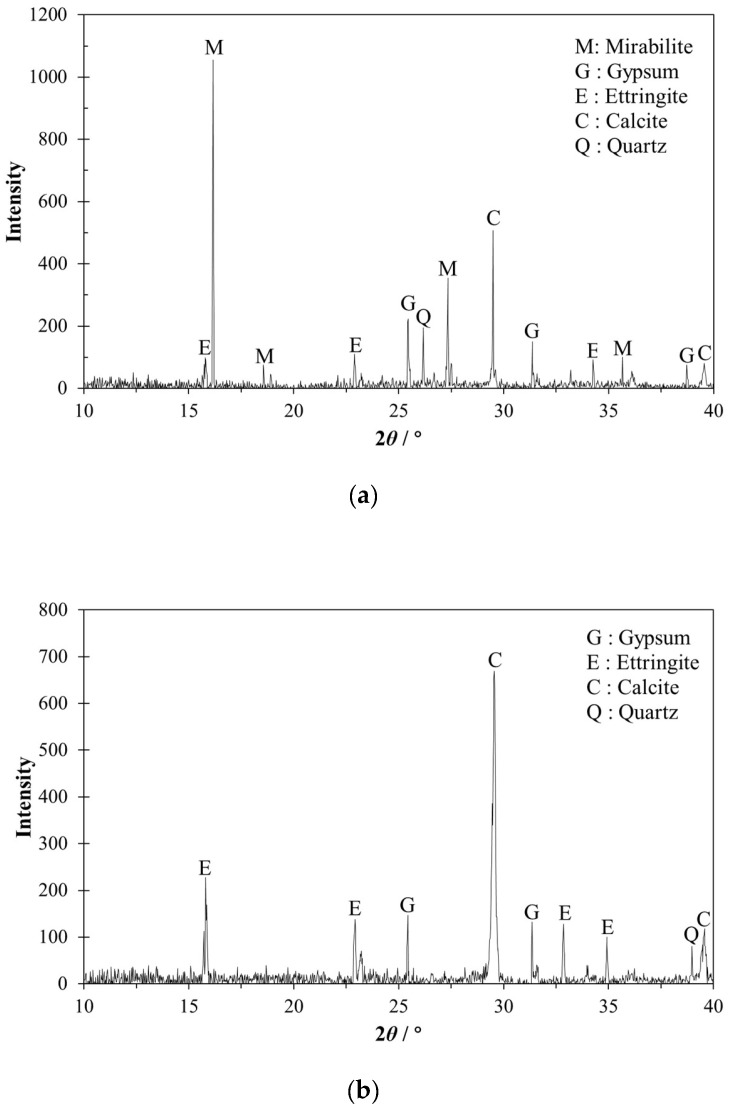
XRD patterns of specimens under different exposure conditions for 90 d: (**a**) Group B undergoing sulfate attack; (**b**) group C cured in distilled water.

To verify the reasons for the variation of the mass of structural EPS concrete, XRD analysis was carried out on the structural EPS concrete subjected to different exposure conditions for 90 days. As shown in Figure 10a, the XRD (Smart Lab, Tokyo, Japan) pattern of structural EPS concrete suffering from W-D cyclic sulfate attack exhibited a high intensity of mirabilite, which could fill up the pores in the specimens efficiently. As observed in Figure 10b, ettringite and gypsum were also generated in EPS concrete cured in distilled water, both of which were also products of hydration of cement in addition to sulfate attack. It could be found from Figure 10 that a relatively large amount of calcite was detected in EPS concrete under both exposure conditions. Because the calcium hydroxide inside the specimen took action with carbon dioxide in the air, the calcium carbonate became the main component when XRD analysis was conducted.

**Figure 11:**
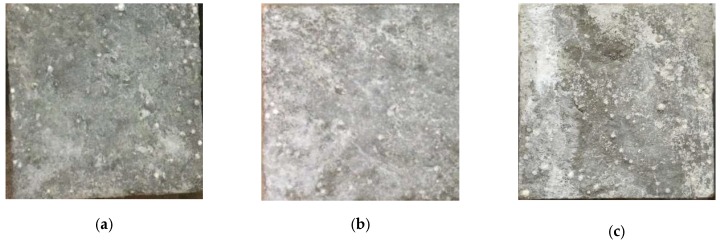
The surface of structural EPS concrete of group B after W-D cycles: (**a**) 90 cycles; (**b**) 120 cycles; (**c**) 150 cycles.

The surfaces of structural EPS concrete of group B after 90, 120, and 150 W-D cycles are illustrated in Figure 11. It could be clearly seen from the picture that more product was generated with the variation of the sulfate attack W-D cycles.

#### 3.2.3. Evolution of Compressive Strength of Structural EPS Concrete

**Figure 12:**
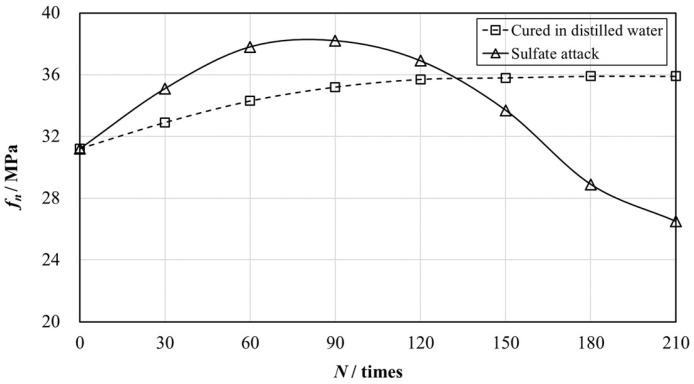
The evolution of the compressive strength of structural EPS concretes of group B and C with different numbers of W-D cycles (*N*).

The evolution of the compressive strength of structural EPS concrete is depicted in Figure 12. As shown in Figure 12, the compressive strength of structural EPS concrete of group B reached the peak value at the cyclic number of 90, while that of structural EPS concrete of group C grew along with the experiment time, monotonously.

After 28-day age, hydration reactions went on slowly inside the concrete specimens, which enhanced the cementation effect between mortar and EPS particles, as well as making the interior space of the specimen denser, contributing to the continued strong growth of samples of group C. It was also found that the compressive strength of samples of group C tended to be stable at the corresponding time of the 120th W-D cycle, which meant that the hydration reaction was nearly completed.

As for the structural EPS concrete of group B, both the hydration reaction and sulfate attack had an effect on the compressive strength. At an early age, products of both the hydration reaction and sulfate attack made the samples denser, resulting in an increase of the compressive strength. However, the hydration reaction approximately finished later, while products of the sulfate attack began making the concrete specimen disintegrate, which led to the decrease in compressive strength.

The level of sulfate resistance of structural EPS concretes prepared in this study was over KS150, which was the highest level regulated in GB/T 50082 [30]. Compared to normal plain concretes, it was attributed to three factors: Addition of fly ash, low water:binder ratio, and incorporation of EPS beads. 

Addition of fly ash could improve the compactness and thus reduced the permeability of the concretes. In addition, the fly ash could alleviate damage from the sulfate attack by improving the interfacial transition zone characteristics [25].

It was pointed out that there was a ‘‘safe zone’’ for concrete with a w/c ratio lower than 0.45 when suffering from sulfate attack [19]. According to the complete hydration rule of cement, the required water was only about 25% by the weight of cementitious materials. However, a relatively large water:binder ratio is commonly used to obtain the necessary liquidity and meet the construction requirements. After the hardening of concretes, the excess water evaporates, and capillary pores are formed. This means that the greater the water:binder ratio is, the greater the permeability coefficient after the cement hydration. Hence, the water:binder ratio of 0.29 employed in the preparation has a positive effect on sulfate resistance of concretes.

Incorporation of EPS beads was the largest difference of structural EPS concrete from plain concrete. It is well known that EPS beads have a superior deformation performance, which could make the concrete capable of bearing greater amounts of products of multiple reactions in both physical and chemical attacks. This effect and the influence of the other factors mentioned before made the sulfate resistance of structural EPS concrete more outstanding than normal plain concrete, though higher moisture migration and absorption capacity might be induced by the incorporation of EPS beads [2].

#### 3.2.4. Relationship between the Evolution of Compressive Strength and Variation of Mass

Both the variation of compressive strength and mass of structural EPS concretes under sulfate attack were related to the compactness in the concrete. The dimensionless parameter, Δ*f*, was defined to describe the evolution of compressive strength:

(7) $$\Delta f=(f_{nB}-f_{0})/f_{0},$$

where $f_{0}$ is the initial compressive strength of EPS concrete; $f_{nB}$ is the average compressive strength of EPS concrete of group B experiencing *N* times W-D cycles. All dimensions are in MPa.

The relationship between Δ*f* and Δ*m_B_* was studied. 

**Figure 13:**
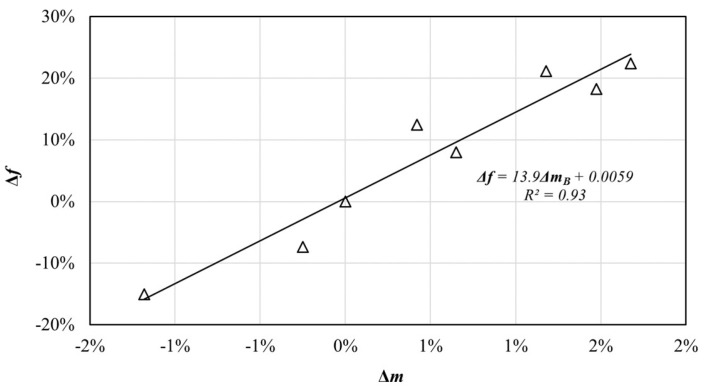
The relationship between Δ*f* and Δ*m_B_* of structural EPS concrete.

As illustrated in Figure 13, these two values showed a linear relationship, with *R*^2^ = 0.93, and their correlation could be expressed as follows:

(8) $$\Delta f=13.9\times \Delta m_{B}+0.0059,$$

where $\Delta m_{B}$ is the variation of mass (Δ*m*) of EPS concrete of group B experiencing *n* W-D cycles.

By Equations (7) and (8), the actual compressive strength of structural EPS concrete under *n* W-D cycles could be predicted by the variation of the mass and initial compressive strength of structural EPS concrete, and no excessive damage would happen to the EPS concrete when measuring the mass variation. The linear relationship between Δ*f* and Δ*m_B_* also indicated that Δ*m_B_* could be substituted for Δ*f* to evaluate the damage degree of structural EPS concrete under sulfate attack because the acquiring method of the indicator, Δ*m_B_*, was more convenient and did not damage the specimen when measuring.

## 4. Conclusions

This paper aimed to explore the fatigue performance and sulfate resistance of structural EPS concrete for long-term service. Thus, long-term cyclic loading and W-D cyclic sulfate attack experiments were conducted on structural EPS concretes with a constant mixture proportion and EPS incorporation ratio. From the experiment results and comparison with the previous literature, the following conclusions can be drawn:

(1) Based on the dynamic stress–strain hysteresis curve of structural EPS concrete under long-term cyclic loading, the dynamic stress–strain curve of structural EPS concrete basically overlapped with each other under the same load and frequency, which indicated that the structural EPS concrete had good stability under a dynamic load.
(2) Compared with plain concretes of the same strength level, the damping ratio of structural EPS concrete was larger, and the dynamic elastic modulus was smaller, which indicated that structural EPS concrete had a superior performance of vibration attenuation, toughness.
(3) A slight crack occurred on the surface of the concrete specimen at about the cyclic number of 15.7 million. However, the relevant dynamic properties could remain stable, which proved the fatigue stability of the structural EPS concrete.
(4) The sulfate resistance of structural EPS concrete was evaluated as the highest level regulated by GB/T 50082 [30] owing to the addition of fly ash, low water:binder ratio, and the incorporation of EPS beads. The structural EPS concrete exhibited a much higher sulfate resistance than plain concrete on the basis of the previous literature.
(5) The indices Δ*f* and Δ*m_B_* were presented to evaluate the variation of compressive strength and mass of structural EPS concretes, which could be used to describe the damage extent of structural EPS concrete under sulfate attack. XRD analysis illustrated that the products generated in the specimens were the reasons for the evolution of the compressive strength and mass. There was an approximately linear relationship between these two indices, which indicated that Δ*m_B_* could be substituted for Δ*f* to evaluate the degree of structural EPS concrete damage under sulfate attack for a more convenient acquiring method and no damage when measuring.

As an exploratory study on the properties of structural EPS concretes for long-term service, the type of structural EPS concretes specimens prepared for long-term cyclic loading and W-D cyclic sulfate attack experiments was limited. Further experiments could be carried out to study the influence of mixture proportions and EPS incorporation ratio on the fatigue and durability behavior of structural EPS concretes.

## Figures and Tables

**Table 1:** Properties of fly ash.

Class F Fly Ash	
Chemical Composition (%)	
Silicon dioxide (SiO_2_)	58.27
Aluminum oxide (Al_2_O_3_)	23.65
Ferric oxide (Fe_2_O_3_)	6.28
Calcium oxide (CaO)	4.18
Magnesium oxide (MgO)	1.52
Sulfur trioxide (SO_3_)	0.33
Potassium oxide (K_2_O)	1.95
sodium oxide (Na_2_O)	1.06
Loss on ignition (LOI), %	0.27
*Physical properties*	
Specific gravity	2.20
Blaine (cm^2^/g)	3340

**Table 2:** Mixture proportion of the specimen.

Cement	Fly Ash	Water	Coarse Aggregate (Type A)	Coarse Aggregate (Type B)	Sand	SP	VMA	PE	EPS Volume
(kg/m^3^)	(kg/m^3^)	(kg/m^3^)	(kg/m^3^)	(kg/m^3^)	(kg/m^3^)	(kg/m^3^)	(kg/m^3^)	(kg/m^3^)	(m^3^) ^a^
413	22	126	181	423	534	6.52	0.35	4.35	0.30

^a^ Incorporation volume of EPS per cubic meter.

**Table 3:** Cyclic loading test scheme of structural expanded polystyrene concrete (EPS) concretes.

Stage	Amplitudes of the Bias Force	Frequencies	Accumulated Cyclic Numbers, *n*
Ⅰ	30 kN	5 Hz	0–2,500,000
Ⅱ	40 kN	5 Hz	2,500,000–3,500,000
Ⅲ	40 kN	10 Hz	3,500,000–16,350,000
Ⅳ	30 kN	10 Hz	16,350,000–22,000,000

**Table 4:** Measured compressive strength and *K_f_* of structural EPS concretes.

Cyclic Numbers, *n*	*f_Bn_*/MPa	*f_Cn_*/MPa	*K_f_*
30	35.1	32.9	106.7%
60	37.8	34.3	110.2%
90	38.2	35.2	108.5%
120	36.9	35.7	103.4%
150	33.7	35.8	94.1%
180	28.9	35.9	80.5%
210	26.5	35.9	73.8%

## References

[1] Xu Y., Jiang L., Xu J., Li Y. (2012). Mechanical properties of expanded polystyrene lightweight aggregate concrete and brick. Constr. Build. Mater..

[2] Babu D.S., Babu K.G., Tiong-Huan W. (2006). Effect of polystyrene aggregate size on strength and moisture migration characteristics of lightweight concrete. Cem. Concr. Compos..

[3] Miled K., Le Roy R., Sab K., Boulay C. (2004). Compressive behavior of an idealized EPS lightweight concrete: Size effects and failure mode. Mech. Mater..

[4] Mohammed H.J., Zain M.F.M. (2016). Experimental application of EPS concrete in the new prototype design of the concrete barrier. Constr. Build. Mater..

[5] Liu Y., Ma D., Jiang Z., Xiao F., Huang X., Liu Z., Tang L. (2016). Dynamic response of expanded polystyrene concrete during low speed impact. Constr. Build. Mater..

[6] American Concrete Institute Committee (2014). Guide for Structural Lightweight Aggregate Concrete.

[7] Dima S.O., Sarbit A., Dobre T., Radu A.L., Nicolescu T.V., Lungu A. (2009). The rheological behaviour of lightweight concrete with embedded EPS beads. Mater. Plast..

[8] Chen B., Liu N. (2013). A novel lightweight concrete-fabrication and its thermal and mechanical properties. Constr. Build. Mater..

[9] Ferrándiz-Mas V., Sarabia L.A., Ortiz M.C., Cheeseman C.R., García-Alcocel E. (2016). Design of bespoke lightweight cement mortars containing waste expanded polystyrene by experimental statistical methods. Mater. Design.

[10] Zhang D.Y., Wang L.M., Liang X.P. Steady State Dynamic Performance of the Slab with EPS-Blocks. Proceedings of the International Conference on Structures and Building Materials.

[11] Gao H., Bu C., Wang Z., Shen Y., Chen G. (2017). Dynamic characteristics of expanded polystyrene composite soil under traffic loadings considering initial consolidation state. Soil Dyn. Earthq. Eng..

[12] Zhou Y., Li M., He Q., Wen K. (2018). Deformation and Damping Characteristics of Lightweight Clay-EPS Soil under Cyclic Loading. Adv. Civ. Eng..

[13] Shi W., Miao L., Luo J., Wang J., Chen Y. (2016). Durability of modified expanded polystyrene concrete after dynamic cyclic loading. Shock. Vib..

[14] Haque M.N., Al-Khaiat H., Kayali O. (2004). Strength and durability of lightweight concrete. Cem. Concr. Compos..

[15] Babu K.G., Babu D.S. (2004). Performance of fly ash concretes containing lightweight EPS aggregates. Cem. Concr. Compos..

[16] Ravindrarajah R.S., Tuck A.J. (1994). Properties of hardened concrete containing treated expanded polystyrene beads. Cem. Concr. Compos..

[17] Ghafoori N., Najimi M., Diawara H., Islam M.S. (2015). Effects of class F fly ash on sulfate resistance of Type V Portland cement concretes under continuous and interrupted sulfate exposures. Constr. Build. Mater..

[18] Zhutovsky S., Hooton R.D. (2017). Experimental study on physical sulfate salt attack. Mater. Struct..

[19] Monteiro P.J.M., Kurtis K.E. (2003). Time to failure for concrete exposed to severe sulfate attack. Cem. Concr. Res..

[20] Babu K.G., Babu D.S. (2003). Behaviour of lightweight expanded polystyrene concrete containing silica fume. Cem. Concr. Res..

[21] Diab A.M., Awad A.M., Elyamany H.E., Abd Elmoaty A.E.M. (2012). Guidelines in compressive strength assessment of concrete modified with silica fume due to magnesium sulfate attack. Constr. Build. Mater..

[22] Sawicz Z., Heng S.S. (1996). Durability of concrete with addition of limestone powder. Mag. Concr. Res..

[23] (2017). ASTM C150/C150M–Standard Specification for Portland Cement.

[24] (2015). ASTM C618–Standard Specification for Coal Fly Ash and Raw or Calcined Natural Pozzolan for Use in Concrete.

[25] Bonakdar A., Mobasher B. (2010). Multi-parameter study of external sulfate attack in blended cement materials. Constr. Build. Mater..

[26] You Q., Miao L., Li C., Hu S., Fang H. (2019). Experimental study on preventing expanded polystyrene concrete segregation. Adv. Cem. Res..

[27] Li C., Miao L., You Q., Hu S., Fang H. (2018). Effects of viscosity modifying admixture (VMA) on workability and compressive strength of structural EPS concrete. Constr. Build. Mater..

[28] (2016). ASTM C192–Standard Practice for Making and Curing Concrete Test Specimens in the Laboratory.

[29] (2013). ASTM C 511–Standard Specification for Mixing Rooms, Moist Cabinets, Moist Rooms, and Water Storage Tanks Used in the Testing of Hydraulic Cements and Concretes.

[30] Ministry of Housing and Urban-Rural Development of the People’s Republic of China (2009). Standard for Test Methods of Long-Term Performance and Durability of Ordinary Concrete.

[31] Rahman M.M., Bassuoni M.T. (2014). Thaumasite sulfate attack on concrete: Mechanisms, influential factors and mitigation. Constr. Build. Mater..

[32] Gao H., Shen Y., Wang Z. (2017). Dynamic modulus and damping ratio characteristics of EPS composite soil. Chin. J. Geotech. Eng..

[33] Ke G., Guo C., Hu S., Chen Z. (2004). Study on the damping ratio of concrete. J. Build. Mater..

[34] Jin Z., Cui Z. (2010). Investigation on strain dependence of dynamic recrystallization behavior using an inverse analysis method. Mater. Sci. Eng. A Struct..

[35] Lydon F.D., Balendran R.V. (1986). Some observations on elastic properties of plain concrete. Cem. Concr. Res..

[36] Al-Amawee A.H., Salman M.M. (2006). The Ratio between Static and Dynamic Modulusof Elasticity in Normal and High Strength Concrete. J. Eng. Sustain. Dev..

[37] Flatt R.J. (2002). Salt damage in porous materials: How high supersaturations are generated. J. Cryst. Growth.

[38] Aye T., Oguchi C.T. (2011). Resistance of plain and blended cement mortars exposed to severe sulfate attacks. Constr. Build. Mater..

[39] Haynes H., O’Neill R., Neff M., Mehta P.K. (2008). Salt weathering distress on concrete exposed to sodium sulfate environment. ACI Mater. J..

[40] Liao Y.D., Yang Y.C., Jiang C.H., Feng X.G., Chen D. (2015). Degradation of mechanical properties of cementitious materials exposed to wet–dry cycles of sulphate solution. Mater. Res. Innov..

